# Scoping Review of Pre-Consumption Food Loss in the US Supply Chain: Factors, Impacts, and Solutions

**DOI:** 10.3390/foods15040775

**Published:** 2026-02-21

**Authors:** Shuai Ma, Laxmi Prasanna Kandi, Zhihong Xu, Peng Lu, Kim E. Dooley

**Affiliations:** 1 Department of Agricultural Leadership, Education, and Communications, Texas A&M University, College Station, TX 77843, USA; laxmiprasanna.kandi@tamu.edu (L.P.K.); xuzhihong@tamu.edu (Z.X.); k-dooley@tamu.edu (K.E.D.); 2 Department of Agricultural Leadership, Education, and Communication, University of Georgia, Athens, GA 30602, USA; penglu@uga.edu

**Keywords:** food loss and waste, United States, scoping review, supply chain, machine learning-assisted review

## Abstract

Food waste is a major global problem that worsens food insecurity and contributes to environmental challenges and resource depletion. Reducing food waste, especially before it reaches consumers, is a crucial strategy for combating food insecurity and advancing environmental sustainability. This scoping review examines the factors, impacts, and practices related to food loss and waste (FLW) in the pre-consumption stage of the U.S. supply chain using a predefined coding scheme. A machine learning technique (i.e., topic modeling) was used to supplement the manual coding to identify themes. Findings from 104 articles from 2015 to 2024 revealed that (a) macro and micro-level factors were understudied; (b) impacts of FLW were predominantly assessed in terms of environmental consequences, with less attention given to economic, social, cultural, and political impacts; (c) despite the high concentration on donation, prevention, recovery, and recycling as solutions, there were critical gaps in the exploration of policy and regulatory strategies, as well as education and awareness; and (d) minimization is the most dominant approach compared to prevention. We recommend that more research focus on causes of food loss, economic, social, cultural, and political impacts, policy and regulatory strategies, as well as education and awareness. We also recommend shifting from weak minimization efforts to strong prevention practices, emphasizing cooperation among all participants in the supply chain.

## 1. Introduction

Food insecurity represents a widespread global challenge, with projections from the United Nations indicating that the population globally will increase to 8.5 billion by 2030 and 9.7 billion by 2050 [1]. As the population expands, meeting the nutritional needs of everyone becomes even more difficult. Paradoxically, around one-third of food produced globally is either lost or wasted annually, resulting in the loss of $1 trillion each year [2]. Despite such massive losses, nearly 8.9% of the population worldwide was undernourished in 2019 [3]. Even in developed areas like the U.S., over one-eighth of the population struggles with food insecurity, which poses risks to national health and security [4]. Food waste has remained relatively stable as a proportion of supply since the 1960s globally and since the 1970s in the U.S. [5]. Food waste not only means missing out on resources that could have fed people in need but also adds to serious environmental problems, like greenhouse gas (GHG) emissions and climate change. Agriculture, which consumes the largest share of freshwater globally [6], is resource-intensive, and livestock production contributes significantly to anthropogenic GHG emissions [7]. In the U.S., landfills produce methane emissions, which contribute to climate change, and food waste generates a large portion of landfill waste [8]. Addressing food waste is, therefore, crucial for promoting sustainable agriculture and protecting the environment. Organizations like the US Environmental Protection Agency (EPA), European Commission (EC), and United Nations (UN) are collaborating at the policy level to combat food issues. Reducing food waste is a key approach to enhancing food security and addressing hunger. Food loss and waste represent a major sustainability and food security challenge in the United States. The U.S. generates the largest amount of household food waste among high-income countries [9]. In addition, the United States has emerged as a key policy and institutional actor in efforts to reduce food loss and waste. For instance, since 2015, federal agencies such as the US EPA and the U.S. Department of Agriculture (USDA) have initiated major national programs and plans for reducing food loss and waste (FLW) by half by 2030, supporting the United Nations’ Sustainable Development Goals [10]. In the United States, hundreds of leaders from government, business, and nonprofit sectors convened at events such as the first Zero Food Waste Forum in California in 2014, organized by environmental groups, and the second forum in Texas in 2015, co-led in part by the U.S. Zero Waste Business Council. Those programs, targets, and efforts make the U.S. a particularly relevant context for synthesizing evidence and identifying research priorities. Previous studies have compared cities in the US with India and found key differences between the food systems regarding supply chain and diet, indicating the importance of considering the country’s socioeconomic stages in food waste impacts [11]. As a representative high-income, developed country with a highly industrialized food system, the U.S. context can also provide valuable insights for other countries at similar stages of economic development. Even within the same socioeconomic status, differences still exist among developed countries in food loss and waste. For instance, a comparison study between the US and France found variation, and France was more active in public sector engagement [12]. Those differences with Europe indicate a need for a study solely focusing on the US context.

A significant portion of FLW occurs during the first few stages of the food supply chain (FSC) worldwide [13,14]. FAO reports that the world has made no substantial progress in reducing food losses since 2015 [15], indicating the continuing importance of examining food loss across supply chain stages. In the U.S., nearly 60% of food waste happens before reaching consumers [16]. This underlines the importance of focusing on early stages in the U.S. supply chain. However, few prior reviews have examined these initial stages in detail. In addition, this review aims to provide a broad overview of FLW by analyzing the factors, impacts, and practices involved in the early phases of the supply chain. This study was guided by three research questions:

- RQ1: What are the study characteristics and methodology details?
- RQ2: What are the factors, impacts, and solutions of FLW in the pre-consumption stage?
- RQ3: What are the key themes related to FLW factors, impacts, and solutions identified through machine learning–assisted analysis?

This scoping review makes three key contributions. There is a lack of utilization of machine learning-assisted methods for conducting review studies. This study makes its unique contribution by focusing on the U.S. through methodological innovation. First, methodologically, we integrate machine learning–assisted analysis of abstracts as a complement to traditional manual coding. While RQ1–RQ2 follow the standard scoping review framework, RQ3 extends prior approaches by applying machine learning–assisted thematic modeling to systematically identify emergent patterns across the literature. To our knowledge, this is among the first attempts to apply machine learning–supported review methods in the food loss and waste literature, helping to compare algorithm-generated themes with human-identified patterns. Second, theoretically, we propose a holistic pre-consumption food loss reduction framework that synthesizes major supply chain phases, key drivers, impacts, and solution pathways, offering guidance for future research and practice. Third, practically, the review highlights geographic and regional gaps in U.S.-based evidence, identifies underexamined areas, and suggests a needed shift from downstream minimization toward upstream prevention and source reduction. As a major high-income country, the U.S. context also enables meaningful comparison with European and other developed settings, supporting both generalizability and the identification of context-specific priorities.

## 2. Literature Review

### 2.1. Food Loss and Waste (FLW)

#### 2.1.1. Definition of FLW

Definitions of FLW vary across five broad dimensions: food quality, intended use, human consumption suitability, the stage of the FSC, and the ultimate destination of the food. These varying academic viewpoints highlight the difficulty in reaching a widely accepted definition of FLW [17]. Some researchers do not differentiate between FLW, instead using “food waste” as a general term. For example, Dou et al. [18] define food waste as the volume of food supplied and ultimately thrown away at any stage within the supply chain. Similarly, Bellemare et al. [19] define food waste as anything that is eventually disposed of in a landfill. Conversely, many researchers clearly distinguish the two separate terms based on the early vs. later phase or upstream vs. downstream FSC [20,21,22] or pre-consumption vs. consumption stages [23,24]. The most recent and widely recognized FLW definition comes from the UN Food and Agriculture Organization [25], which differentiates food loss as the reduction in quality or quantity of produce due to decisions made in the FSC, while food waste refers to losses attributed to retailers, catering services, or food establishments, and consumers. For this review, we define food loss as edible and inedible food materials that are removed from the human body pre-consumption food supply chain during harvest/slaughter, on-farm postharvest/slaughter operations, transport storage distribution, processing, and packaging.

#### 2.1.2. FLW Across Supply Chain Phases

There are different categorizations of supply chain phases concerning FLW. Some studies divide the phases into harvesting, processing/manufacturing, wholesale, retail, and consumer stages [26,27]. Conrad [28] simplified them into farm, transportation, wholesale, and retail. Singh and Singh [29] and Porter et al. [22] offer a more detailed structure, which consists of harvesting, after-harvest handling, storage and transport, processing, delivery, and reaching consumers. Minn [30] categorized it into agricultural production, industrial processing/packaging, transportation, wholesale/retail, and household.

FLW stages can also be broadly divided into pre-consumption and consumption stages. Food loss in the pre-consumption stage includes harvest/slaughter, on-farm postharvest/slaughter operations, transport, storage, distribution, and processing and packaging. Consumption stages encompass commercial, public, and domestic households [31,32]. This study focuses specifically on the pre-consumption stages based on this categorization, aiming to reduce FLW at the source. Table 1 demonstrates a summary of the FLW supply chain phases.

#### 2.1.3. Factors, Impacts, and Solutions of Food Lost in the Pre-Consumption Stage

FLW in the pre-consumption stage can take place for various causes, including deliberate overproduction due to the unpredictability of farmers’ decisions influenced by environmental and economic factors [36]. For instance, produce might be left in the field on purpose if it does not meet visual or aesthetic standards [37]. Additionally, unavoidable factors, like spoilage caused by weather or equipment failures during processing, are also significant contributors to food loss [38,39]. The impacts of FLW are multifaceted, including economic, environmental, and social consequences [40]. For instance, losses at the farm level can have an adverse impact on the financial sustainability of growers [41], and the decomposition of waste food in landfills generates GHG emissions [42].

Several solutions to FLW have been identified in the literature, including technological advancement, by-product utilization, and waste-to-energy practice. For example, techniques/methods like heat processing, salting, and acidification can be used to reduce spoilage [43], while waste-to-energy conversion offers a sustainable alternative to petroleum-derived [44].

#### 2.1.4. Food Recovery Hierarchy

The Food Recovery Hierarchy (FRH), organized by the Environmental Protection Agency [45], prioritizes from the most preferred to least preferred: source reduction, feeding needy people, feeding animals, industrial uses, composting, and landfill/incineration. Source reduction entails decreasing the production of extra food. Donating extra food to organizations, food pantries, and shelters to help feed the hungry. Feeding animals means redirecting food scraps to provide food for animals. Industrial uses include converting waste oils into fuel and processing food scraps for energy recovery. Composting transforms food waste into a nutrient-dense soil amendment. Discarding wasted food to landfill is the final and least desired choice for disposal. This hierarchy emphasizes minimizing food surplus, redirecting excess food to those in need, and utilizing waste for energy or composting before considering disposal [45].

In 2023, the EPA updated the hierarchy to the Wasted Food Scale, reflecting technological advancement and operational practices [46].

### 2.2. Previous Literature Review and Research Gap

Previous review studies have provided important overviews of food loss and waste (FLW) research, but they leave several gaps that motivate the present study. Most existing reviews examine FLW broadly across the entire supply chain (e.g., Refs. [32,47]) without offering detailed attention to the early, pre-consumption phases where substantial losses occur. In addition, prior syntheses often concentrate on selected dimensions of FLW factors, impacts, or solutions (e.g., Refs. [17,32,48]) rather than integrating these components into a holistic understanding of upstream loss dynamics. For example, Moraes et al. [48] primarily emphasize FLW reduction practices (prevention and minimization) across food chain operations, but provide a limited focus on factors and impacts. Chauhan et al. [17] identify major themes, including measurement and emerging digital tools and a holistic discussion of factors, impacts, and solutions, yet their scope remains global and does not offer a focused synthesis of pre-consumption stages within a specific national context. Similarly, Özbük and Coşkun [49] focus specifically on downstream food waste and classify contributing factors into internal, micro-, and macro-environmental categories; however, upstream pre-consumption losses remain underexplored. Da Costa et al. [50] adopt a technology-centered perspective on IoT applications, highlighting innovation trends but narrowing attention away from broader determinants of food loss.

Some previous studies have a global perspective [48,49,50], and some examine a specific region/country [32,47]. For instance, Li et al. [32] investigate FLW throughout the supply chain in China. They looked at quantities of FLW, assessed impacts, and identified factors like food loss mediators (such as supply chain stages) and food waste mediators (cultural factors, societal factors, personal factors, and behavioral factors). The study provides some research gaps, such as date-related issues, a lack of comprehensive interventions at the consumer level, and insufficient focus on behavioral variables. El Bilali et al. [47] examine FLW in the western Balkans throughout the supply chain; the present scale of FLW and the causes, impact, and management strategies. For studies with a specific country/region, both of the two reviews focus on developing countries/regions, and there is a lack of focus on developed countries, which represent different trends and practices due to different economic statuses and technology advancement levels.

None of the existing reviews have concentrated solely on the U.S., highlighting a critical gap that this study intends to bridge. By analyzing the factors, impacts, and practices of FLW during pre-consumption stages in the U.S., this review aims to provide an in-depth understanding of the current research landscape, pinpoint existing gaps, and outline future directions. It seeks to address pivotal questions about what, why, how, and where FLW can be mitigated, thereby strengthening food and agricultural security on both national and global scales.

In addition, existing review studies in the food loss and waste context used traditional manual coding to conduct systematic reviews and lacked methodological innovation. Previous studies have explored utilizing machine learning-assisted reviews. For example, Xu et al. [51] explored machine learning methods and large language models for assisting the title and abstract stage in systematic reviews. Topic modeling is a text-mining technique commonly used in machine learning and natural language processing. Verma & Yuvaraj [52] generated themes through topic modeling-assisted review using a title and abstract dataset, indicating its effectiveness. This study aims to make a contribution to the food waste literature by employing machine learning techniques to assist in scoping review. This innovative application of methodology helps reduce researcher bias for systematic review [52].

## 3. Theoretical Framework

**Figure 1:**
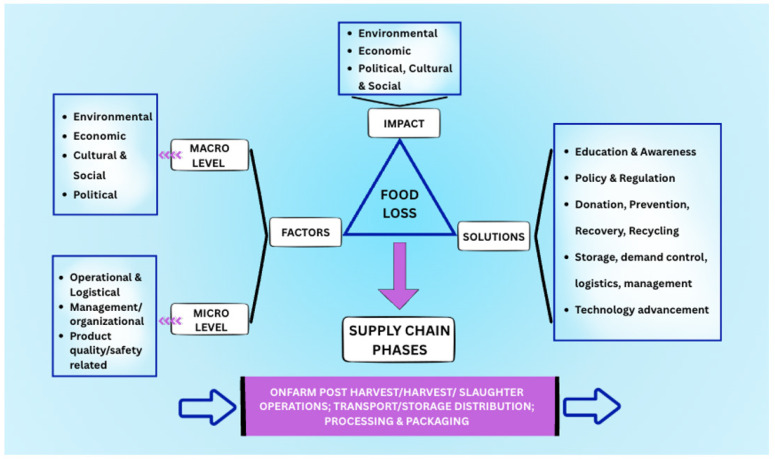
Food Loss Reduction Model in the pre-consumption stage. **Note.** Arrows indicate directional relationships among factors, supply chain phases, and proposed solutions influencing food loss across macro and micro levels.

Building on the categorization of factors by Li et al. [32] and Özbük and Coşkun [49], Urbaniak et al.’s [40] impact classification, and Moraes et al.’s [48] categorization of solutions, the theoretical framework integrates these components with supply chain phases [31,32]. As demonstrated in Figure 1, the Food Loss Reduction Model in the pre-consumption stage unites the “what, why, how, and where” of FLW. This holistic approach aims to determine the most sustainable practices to minimize food loss, supporting the overarching goals of food security and sustainable development.

## 4. Methods

### 4.1. Search Strategy

An in-depth search strategy was used, focusing on three key concepts: food loss and waste, the food supply chain, and the U.S. For each concept, synonyms, related terms, and database subject headings were identified and combined using the Boolean operator OR to form a search cluster. For instance, the words for the concept of FLW are “Food wast*,” “food loss*,” “food surplus*,” “food discard,” and “food spoil*.” The words of the concept for pre-consumption supply chain are “supply chain,” “supply chains,” “logistic*,” “postharvest*,” “manufactur*,” “distribut*,” “process*,” “industr*,” “stor*,” “harvest*,” “slaughter*,” “transport*,” “packag*,” and “product*.”

The search was developed in databases like Agricola (EBSCO), the FSTA (EBSCO), and CAB Abstracts (EBSCO). We provided a detailed search strategy in the supplemental materials for three databases. The article search publishing timeframe was from January 2015 to March 2024, and all the searches were conducted in March 2024.

### 4.2. Selection Criteria

The selection criteria for studies included in this review underscore the review’s commitment to rigorously investigating the factors, impacts, and solutions of food loss in the pre-consumption stage across early FSC stages in the U.S. Since 2015, the USDA and EPA have introduced a plan to reduce significant FLW, supporting the United Nations’ Sustainable Development Goals [8,45]. ReFED was established in 2015 as a joint effort involving over 30 leaders from industry, nonprofits, foundations, and government, all dedicated to decreasing food waste in the U.S. [53]. We chose the date of 2015 as a time cutoff since several efforts have been made, and goals have been set by government and nonprofit organizations in 2015; FLW in the US gained attention and focus after 2015. The criteria are summarized as follows:

Inclusion:

- The studies must be in the U.S.
- The studies must include one or more supply chain stages in the pre-consumption phase: harvest/slaughter, on-farm postharvest/slaughter operations, transport/storage distribution, and processing and packaging.
- The studies must be on food loss/waste-related topics.
- The studies must be written in English.
- The studies must be published from 2015 to 2024.
- The studies must be peer-reviewed journal articles, conference proceedings, and dissertations/theses.
- Studies must mention one or more of the components: factors, impact, and solution of food loss/waste.
- Studies that have mentioned the U.S. but also talk about other countries from a global perspective.

Exclusion:

- Books, book reviews, and reviews of theoretical and conceptual studies were excluded.
- Studies that examine consumption (retail, public, and household consumption) were excluded. (e.g., hotel, wholesale, grocery, food service, school, university, organization).
- Post-consumption food waste was excluded.
- Studies focusing solely on recycling or reusing animal waste (manure) were excluded.

### 4.3. Coding Scheme

#### 4.3.1. Study Characteristics

Study characteristics include publication year, state, article type, journal name, conference name, research method, research type, food category, and FSC phase. The year was recorded as the year of the publication, and the state refers to the state in the US where the study was conducted. Studies that did not report that information were coded as not mentioned. Studies that were conducted in multiple states were coded as mixed. Article types were divided into journal articles, conference proceedings, and dissertations/theses. The journal name and conference name were coded to indicate where it was published/presented. Research methods were divided into qualitative, quantitative, and mixed methods.

**Research type**: Research types were divided into modeling-based quantitative analysis (e.g., economic simulation/modeling, environmental modeling), intervention/experiment study, second-hand data analysis, case studies/tutorial cases, survey/interview empirical study, and theoretical/conceptual paper.

**Food category**: The food categories were grains and cereals, vegetables and fruits, dairy, meat and poultry, aquatic foods, other (legumes and oilseeds, sweeteners and byproducts, alcoholic beverages, nuts), and mixed.

**Food supply chain phase (pre-consumption stage)**: The FSC stages in this review were divided into harvest/slaughter, on-farm postharvest/slaughter operations, transport, storage, distribution, processing, and packaging [31,32]. If more than one stage is involved, it is coded as mixed.

#### 4.3.2. Factors and Impacts

**Factors**: According to Li et al. [32], factors can be divided into culture factors, societal factors, personal factors, and behavioral factors. Özbük and Coşkun [49] define internal factors as organizational, operational, marketing-related, product-related factors and handling practices, and macro-environmental factors like natural, political, economic, and sociocultural factors. Based on these frameworks [32,49], we defined factors as macro-level and micro-level factors. Macro-level factor includes environmental (weather, pests, plant disease, high temperature), economic (economic cost, economic losses, cost–benefit that influence farmers’ decision), cultural and social factors (food recovery partnerships, traditional food waste handling practices), political factor like policy and regulation (food policy, geopolitical border, labeling practice of best before date, restricting nutritional factor into animal feed), and mixed. Micro-level factor includes operational and logistic/operational factor, management/organizational factors (poor handling practices, challenges in the distribution network, issues in the donation program), product quality/safety-related factors (poor control over microbial food spoilage, internal decay, microbial contamination, inefficient cleaning practices), and mixed.

**Impacts**: Urbaniak et al. [40] divided impact into social impact, technical, environmental, economic, and political impact. Based on this framework, we divided impact into environmental impact, economic impact, social impact, cultural impact, and political impact. If more than one impact is involved, it is coded as mixed.

#### 4.3.3. Solutions

**Solutions**: Moraes et al. [48] categorized solutions as education and awareness, laws, donations and reuse, research and support, storage, demand, logistics, and marketing efforts. Adopted from this framework by Moraes et al. [48], we categorize solution as education and awareness; policy and regulation; donation/redistribution, prevention (byproduct utilization/upcycling and repurposing); recovery (offer food to hungry people), recycling (provide food to animals, industrial uses, composting); storage, demand control, logistics, management; and technology advancement (e.g., food preservation and shelf-life extension; blockchain for tracking, data collection, using high tunnel, mechanical separation techniques to improve production and handling). If more than one solution is involved, then it is categorized as mixed.

**Food Recovery Hierarchy (FRH)**: Based on the U.S. EPA [35], it serves as a guiding rationale for our categorization. Articles previously coded under solutions such as donation, prevention, recovery, and recycling were further evaluated according to this hierarchy. The FRH prioritizes actions from source reduction. Landfilling is the least desired option. This approach allows us to assess current practices at a micro level, emphasizing the most sustainable methods for reducing food loss. Articles involving more than one hierarchy are coded as mixed.

**Practice evaluation**: Moraes et al. [48] also divided practice and method into prevention and minimization. Prevention methods focus on the impact on the food chain prior to the production/purchase/preparation phases. Minimization methods address actions following the production/purchase/preparation phase, such as reusing leftovers or donating to food banks. In this review, we focus specifically on solutions and industry practices that align with the FRH, like redistribution/donation, prevention, recovery, and recycling. This targeted approach emphasizes sustainability practices that are beneficial for the environment. We divided the practice evaluation into prevention (e.g., any source reduction practices) and minimization. If both prevention and minimization are involved in the paper, we coded it as mixed.

### 4.4. Data Collection and Analysis

**Figure 2:**
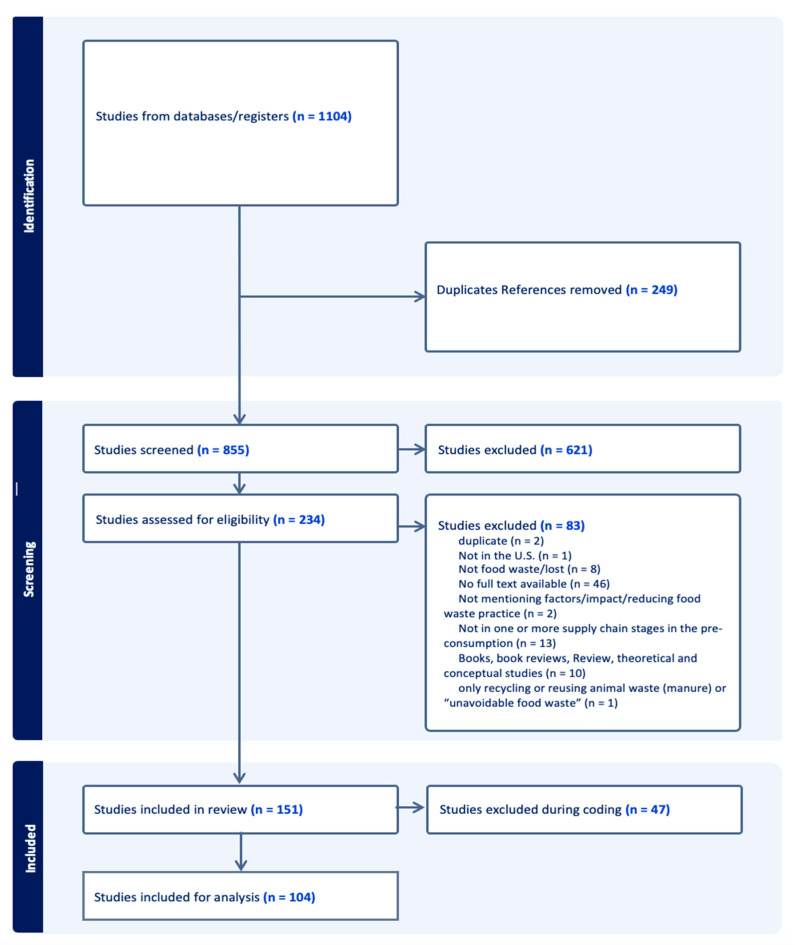
PRISMA flow diagram.

As demonstrated in Figure 2, we initially removed 249 duplicate references and included 855 unique titles and abstracts for title and abstract screening. Two researchers were involved in titles and abstracts screening following the developed inclusion and exclusion criteria, which left 234 references eligible for full-text screening. Two researchers independently reviewed each full-text article, and any conflicts would be resolved through group discussion. After full-text screening, we identified 151 eligible articles for review. There were 47 articles excluded during the coding process; the number of eligible articles for data analysis was 104.

The included studies were coded by two researchers using Microsoft Excel. They initially coded ten articles together in group meetings and then conducted a second round of group coding for five additional articles to ensure consistency. They reached an average inter-rater reliability of 91.38%. After establishing a common understanding, each researcher coded the rest of the articles separately. After all the articles were coded, one coder checked 30 selected articles that were done by a different coder. Finally, descriptive statistics and content analysis were employed to analyze the data.

**Figure 3:**
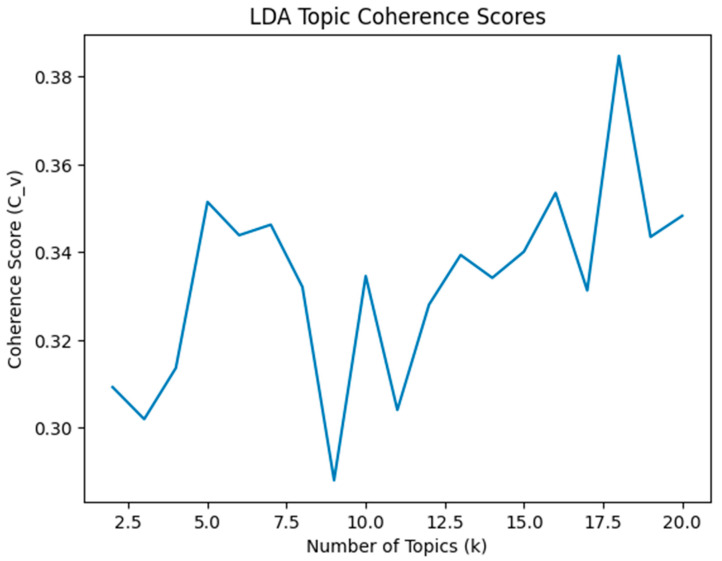
LDA topic coherence scores.

For the machine learning-assisted part, we downloaded the included studies with abstracts from Covidence. Topic modeling, as an unsupervised method, can help clusters of words that categorize a set of documents in the food waste context [54]. Following the common procedures for topic modeling recommended by Wusylko et al. [55], we conducted data preprocessing, removing noise such as punctuation and semantically meaningless stop words. We applied Latent Dirichlet Allocation (LDA) as the topic modeling algorithm for analyzing the data in Google Colab using version Python 3.12.12 [56]. Coherence testing was conducted to help determine the optimal number of topics [57]. Coherence scores range from 0.29 to 0.38. Figure 3 presents the LDA topic coherence scores. Topic coherence scores were relatively stable across a range of topic numbers (*k* = 2–20). The final number of topics (*k* = 5) was selected based on a balance between coherence, interpretability, and alignment with predefined coding schemes. Topic modeling results were qualitatively interpreted and synthesized into higher-level themes based on the most probable words and representative documents. For topic interpretation, the top 50 most probable keywords per topic were examined. To enhance interpretability, we selected representative keywords per topic to report.

## 5. Result and Discussion

### 5.1. Study Characteristics

**Figure 4:**
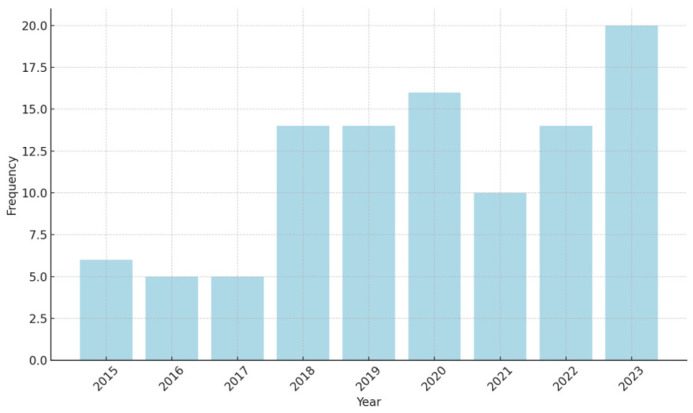
Distribution of years.

Year: Our time frame was limited to 2015 to 2024 in this study. Among 104 studies, 42.31% (*n* = 44) of studies were published from 2015 to 2019, while 57.69% (*n* = 60) emerged during 2020–2024. This indicates a growing interest in the last 5 years. As shown in Figure 4, 19.23% (*n* = 20) of the studies were published in the year 2023 and have the highest frequency. In a systematic review of Chauhan et al. [17], findings indicated a trend of growing increase in academics since 2013, which aligns with our findings.

Article type: As shown in Table 2, journal articles were the most frequent emerging, which make up 88.46% (*n* = 92) of the total articles. This indicates a strong preference for journal publications among researchers. Conference proceedings were the second most frequent, representing 6.73% (*n* = 7). Dissertations and theses were the least common, with only 5 occurrences, accounting for 4.81% of the total.

Journal: This review included a total of ninety-two journal articles. The most frequently cited journals are *Journal of Cleaner Production, Sustainability, Environmental Science & Technology, Renewable and Sustainable Energy Reviews, Journal of Agriculture, Food Systems, and Community Development, Scientia Horticulturae, Fermentation, Resources, Conservation & Recycling.* Notably, 59 journals appear only once or twice, accounting for 56.73% of the total. The included articles strongly emphasize sustainability, environmental science, and clean production.

Conference: Seven studies were presented at conferences. The conferences listed include: ASCPC Conference, Southeast Conference, V International Conference Postharvest Unlimited, Second International Conference on Agriculture in an Urbanizing Society, and 12th International Working Conference on Stored Product Protection (IWCSPP).

**Figure 5:**
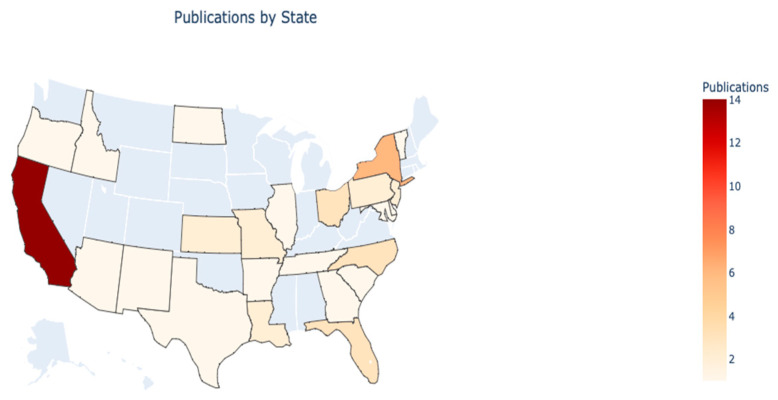
Distribution of states.

State: Figure 5 illustrates the distribution of publications by state across the U.S. The data reveal a strong concentration in California (13.46%, *n* = 14) and New York (5.77%, *n* = 6), with multiple states accounting for a significant portion (25.00%, *n* = 26) of the publications. The color gradient in the figure effectively highlights the varying levels of publication activity, with darker shades indicating a higher number of publications.

The research concentration of publications on California and New York may be influenced by state policy and regulation. For example, California and New York City have laws requiring the business sector to divert food waste from landfills to support zero-waste goals [58], which might increase the interest in industry and academia.

**Research methods:** 82.69% (*n* = 86) of the articles used quantitative research, revealing a dominant preference for quantitative methods. Qualitative methods were employed 12.50% (*n* = 13), while the percentage of mixed methods was 4.81% (*n* = 5). This distribution highlights the dominant role of quantitative research, with qualitative and mixed methods being significantly less utilized. This finding aligns with Chauhan et al. [17], who reported that most of the included studies were quantitative. Future research is encouraged to employ qualitative or mixed methods to gain deeper insights.

**Research type:** Among the included studies, intervention/experiment studies constitute 39.42% (*n* = 41) of the total research types. Survey/interview empirical studies were the next most common, making up 20.19% (*n* = 21). Modeling-based quantitative analysis accounted for 17.31% (*n* = 18), followed by secondhand data analysis at 13.46% (*n* = 14). Case studies/tutorial cases represented 6.73% (*n* = 7), while theoretical/conceptual papers were the least frequent, at 2.88% (*n* = 3). This aligns with findings in a systematic review of downstream food waste by Özbük and Coşkun [49], stating that most of the studies were empirical studies. Özbük and Coşkun [49] also stated that there was a lack of a case study approach, and few studies were conceptual, similar to our findings. More than half of the studies were empirical studies using either experiments or survey designs, providing foundations to potentially do a systematic review/meta-analysis of those practice studies and evaluate the effect of the practices.

**Figure 6:**
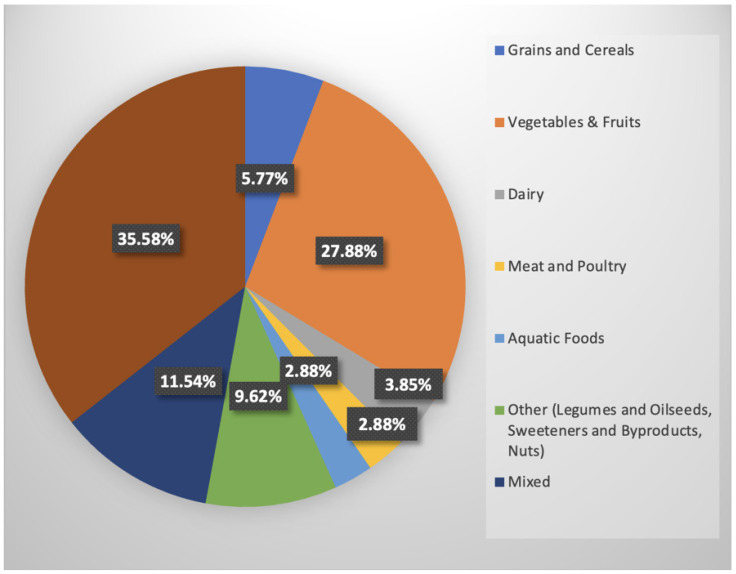
Food categories. Note. Brown indicates “not specified.”

**Food category:** This distribution (Figure 6) indicates that the most frequently analyzed food category was not specified, accounting for 35.58% (*n* = 37) of the total. Among the specified categories, vegetables and fruits were the most prevalent, representing 27.88% (*n* = 29). Other categories, which included legumes, oilseeds, sweeteners, byproducts, alcoholic beverages, and nuts, account for 9.62% (*n* = 10). Mixed categories represented 11.54% (*n* = 12). Less than a quarter of the studies (15.38%, *n* = 16) were associated with the remaining categories (grains and cereals, dairy, meat and poultry, and aquatic foods), which have lower frequencies.

The distribution of studies across different food categories and supply chain phases highlights several knowledge gaps in the current research on FLW. Vegetables and fruits were the most frequently studied categories, accounting for a substantial portion of the research. This focus aligns with the high rates of FLW in these categories, particularly in North America, where 50–60% of fruits and vegetables were lost from food in the supply chain [20]. It might also be associated with the predominant studies carried out in California, which ranks as the top producer of vegetables and fruits in the United States. Other food categories, such as grains, cereals, dairy, meat, and poultry, also contributed significantly to FLW. We suggest that future research include these additional categories to broaden our knowledge of the entire spectrum of food loss and to address the unique challenges linked to various types of food products.

**Figure 7:**
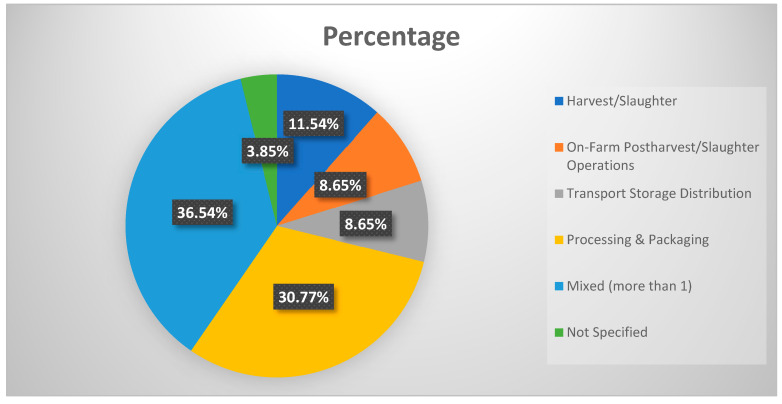
Supply chain phase.

**Supply chain phase**: The distribution (Figure 7) demonstrated that 36.54% of the studies involved the “mixed” phase, which was the most frequent. Processing and packaging followed with 30.77%, indicating a significant focus on this stage of the supply chain. Harvest/slaughter constitutes 11.54%, while on-farm postharvest/slaughter operations and transport, storage, and distribution each account for 8.65%. A small portion of the entries, 3.85%, do not specify the supply chain phase. This is in line with previous studies stating that only a limited amount of surplus food on the farm is recovered in the U.S., and FLW at this stage remains a largely underexplored area of research [59].

Moreover, the analysis of supply chain phases reveals that most studies focus on the processing and packaging stages, with fewer studies addressing earlier phases such as harvest/slaughter and post-harvest operations. This imbalanced emphasis on the later stages of the supply chain can be attributed to the measurable nature of waste at these stages. However, significant losses take place in the early stages, particularly at harvest and on-farm post-harvest loss, where issues like the cost of harvesting and laboring issues for the harvest stage affect the farmer’s decision-making process and operational factors, environmental factors, and socioeconomic factors for post-harvest contribute to substantial losses [60]. The lack of attention to these early stages suggests that future research should prioritize understanding and addressing FLW at the point of origin, where interventions could prevent losses before they propagate through the supply chain. Additionally, EPA [10] estimated that the manufacturing and processing sector accounts for almost 39% of the food waste in the U.S. The significant focus on “processing & packaging” in the current research is in alignment with the substantial volume of waste produced at this stage. Future research can focus more on the harvest/slaughter stage as well as on farm postharvest/slaughter operations, with continuous effort on the processing and packaging stage. Future research should also explore how different food categories interact with various supply chain stages to create unique patterns of loss and waste.

### 5.2. Factors and Impacts

**Macro-level factor:** A significant portion of the publications, 84.62% (*n* = 89), do not specify the macro-level factors influencing FLW. Macro-level factors (e.g., policy incentives, market structures, regulatory conditions) are often more difficult to operationalize and measure within empirical studies than operational or technological loss metrics. Operational and measurement constraints may partly explain the limited attention to macro-level factors in the existing literature. Among the specified factors, political factors (policy, regulation, geopolitical, labeling practices) were the most frequently mentioned, accounting for 4.81% (*n* = 5). Economic factors (cost, losses, cost–benefit influencing farmer decisions) influencing farmer decisions, represented 3.85% (*n* = 4) of the studies, while environmental factors (weather, pests, plant disease, high temperature) constituted 1.92% (*n* = 2). Cultural and social factors, though critical, were significantly underrepresented, constituting only 1.00% (*n* = 1) of the studies.

The insufficient focus on macro-level factors in the literature reveals substantial knowledge gaps. Political factors, such as policies and regulations, emerge as the most studied among the specified categories, consistent with findings from systematic reviews like that of Li et al. [32], which emphasized the growing importance of regulatory factors. For instance, Charlebois et al. [61] suggested modifications to food labeling practices, like eliminating “Best Before” dates on some products, to reduce confusion among stakeholders and help cut down on food waste. However, important cultural and social factors that influence food waste behavior were mostly ignored. Studies like Thyberg and Tonjes [62] highlight how cultural perceptions of edibility can significantly influence what portions of food are discarded during the manufacturing stage. For instance, in the U.S., cultural norms lead to the disposal of parts like organ meats and cartilage, which may be considered edible in other countries [63]. The mixed category, encompassing studies that address multiple macro-level factors, also accounts for 3.85% (*n* = 4) of the publications, indicating a prevalence of unspecified macro factors, with political and economic influences being the most frequently addressed.

**Figure 8:**
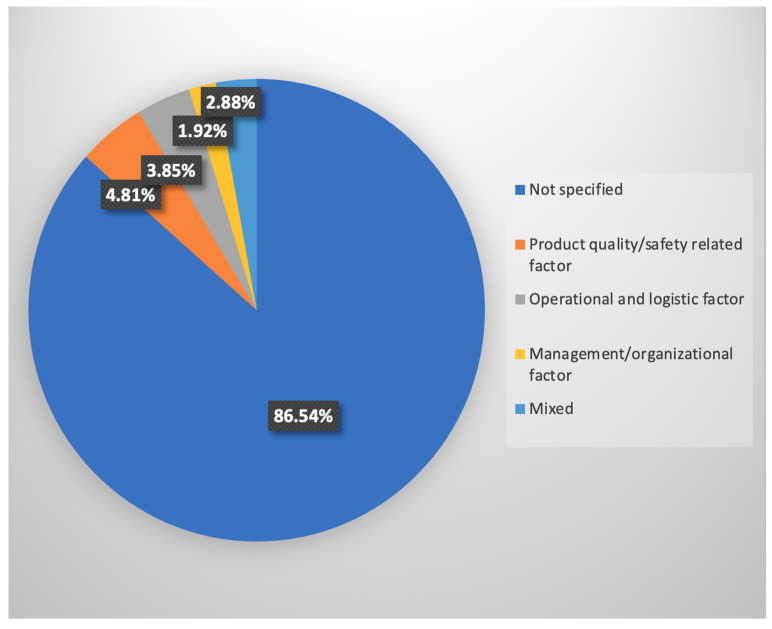
Micro-level factors.

**Micro-level factor:** Figure 8 illustrates that 86.54% (*n* = 90) of the studies did not specify the micro-level factors related to FLW. Micro-level behavioral and organizational factors frequently require primary data collection from producers, processors, and intermediaries, which can be resource-intensive and may face access barriers. This gap reflects not only an absence of research attention but also methodological, institutional, and data-related challenges that shape the U.S. literature. Among those that do, product quality/safety-related factors were the most frequently discussed, accounting for 4.81% (*n* = 7). Operational and logistic factors follow with 3.85% (*n* = 4), and management/organizational factors constitute 1.92% (*n* = 2). The “mixed” category, which included multiple micro-level factors, accounts for 2.88% (*n* = 3).

The low percentage of studies addressing micro-level factors suggests an underdeveloped understanding of the practical causes of FLW, particularly in terms of supply chain efficiency. The intricate nature of supply chains and the participation of numerous stakeholders may make identifying these causes challenging. Operational inefficiency, such as poor handling and storage, can accelerate spoilage and lead to significant food losses. Overall, the current literature underrepresents these micro-level factors, indicating a need for more focused research in this area.

**Figure 9:**
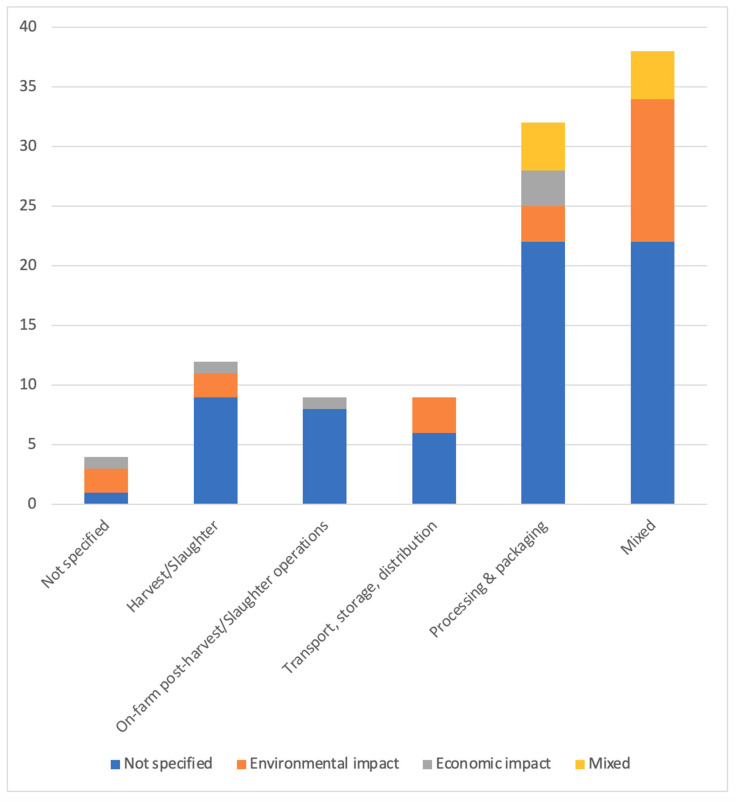
Distribution of impacts across supply chain phases.

**Impacts:** The distribution of studies addressing the impacts of FLW (see Figure 9) revealed that 65.38% (*n* = 68) do not discuss impact. Among the specified impacts, environmental impacts were the most frequently examined, accounting for 21.15% (*n* = 22). Economic impacts followed with 5.77% (*n* = 6). There were no entries categorized as addressing direct social, cultural, or political impact. The “mixed” category, which included multiple types of environmental and economic impacts, accounted for 7.69% (*n* = 8).

The primary emphasis on environmental impacts, such as greenhouse gas emissions and resource depletion, limits our understanding of the broader consequences of FLW. For example, Read and Muth [64] assessed environmental impacts through interventions like global warming and water use. Kuo and Dow [65] discussed the positive and negative impacts of converting food waste into biogas, noting that while this practice reduces greenhouse gas emissions, it also produces air pollutants. Li et al. [32] highlighted the indirect political and social impacts of reducing the reliance on imported food, which enhances food security in China. The limited focus on environmental impacts indicates that future research should take a more comprehensive approach, incorporating financial, cultural, and other dimensions of FLW as well.

### 5.3. Solutions

Solutions: The literature revealed a diverse range of solutions aimed at reducing FLW, with a significant focus on donation, prevention, recovery, and recycling (54.00%, *n* = 38). A quarter of the studies (25.00%, *n* = 26) do not specify the type of solution. Solutions involving storage demand control and logistics management, technology advancement, and mixed solutions each account for 10.58% (*n* = 11) of the dataset. Policy and regulation solutions make up 5.77% (*n* = 6), while education and awareness solutions were the least represented, with only 0.96% (*n* = 1).

Practical and Actionable Solutions: The literature extensively discussed practical and actionable solutions such as donation, prevention, recovery, and recycling, which were essential for addressing FLW. Moraes et al. [48] pointed out that these solutions include byproduct utilization, upcycling, and repurposing, which align with prevention; donation and redistribution, which fall under recovery; and recycling practices like feeding animals, industrial uses, and composting. Additionally, product utilization and upcycling are emerging areas of interest. Stoklosa et al. [66] introduced the use of byproducts like butyric acid produced from sweet sorghum bagasse (SSB), highlighting its potential for biofuels and high-value chemicals. Aita et al. [67] mentioned the byproduct fumaric acid produced from cane bagasse. A lignocellulosic syrup, treated with powdered activated carbon and derived from energy cane bagasse, was utilized to obtain this byproduct as a potential feedstock. Dorado et al. [68] discussed byproducts from Citrus sinensis (orange) juice processing for extracting sugars, peel oil, and other valuable compounds. These practices not only reduce waste but also create economic value.

Gaps in Policy, Regulation, Education, and Awareness: Despite the concentration on donation, prevention, recovery, and recycling as solutions, critical gaps remain in the exploration of policy and regulatory strategies, along with educational and awareness initiatives. Policy and regulation, as noted by Yan et al. [69], can establish standards, monitor practices, and enhance sustainability awareness among stakeholders, creating systematic changes that reduce FLW across the supply chain. However, these areas are underrepresented in the current literature. Education and awareness were particularly critical in shifting consumer and industry behaviors toward more sustainable practices. Gosliner et al. [70] showed that multisector partnerships are effective in raising awareness about food waste, indicating that educational initiatives can play a significant role in reducing FLW.

The Role of Technology Advancement: Technology advancement is another critical component in reducing FLW. Solutions in this category include food preservation techniques, shelf-life extension, and innovations like blockchain for tracking and data collection. For example, Vincent et al. [2] discussed how blockchain technology can improve food security by enabling precise tracking during food recall scenarios, thus reducing waste. Similarly, Begum et al. [71] explored the development of technology to extend the shelf life of strawberries using advanced packaging materials, showcasing how technological innovations can mitigate food spoilage and loss.

**Figure 10:**
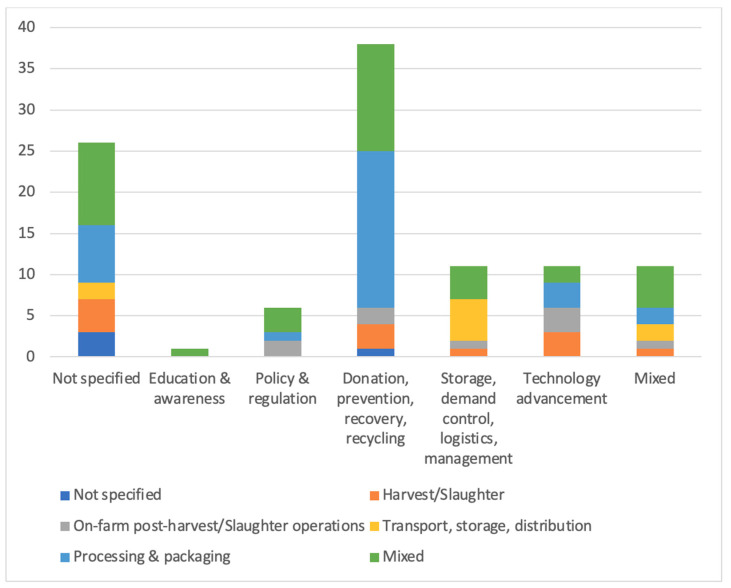
Solutions across supply chain phases.

Figure 10 demonstrated the distribution of solutions across supply chain phases, indicating a high concentration on donation, prevention, recovery, and recycling as solutions in the processing and packaging stage, but with a lack of attention in the early stages before processing and packaging. Addressing FLW requires open and effective collaboration among stakeholders across the entire FSC, yet this is another area where existing research is limited. The literature suggests that while there were efforts to engage different stakeholders, such as through food recovery partnerships or industry collaborations, achieving the goal of reducing food waste was challenging because of the numerous stakeholders, food-related industries, and the multiple points where resources are wasted [72]. There is a need for research that examines how stakeholders, including farmers, producers, distributors, retailers, and consumers, can collaborate to minimize food waste more effectively. Furthermore, the role of government and policymakers in facilitating stakeholder collaboration is critical. Policies that incentivize collaboration, such as the Nutrition Policy Institute and the Public Health Alliance of Southern California, launched a collaborative effort across multiple sectors involving California state agencies. Excessive waste could be instrumental in bringing different sectors together [70]. Further research needs to emphasize the development and evaluation of models of stakeholder collaboration that are adaptable to different contexts within the U.S. food system.

Food Recovery Hierarchy (FRH): The FRH, based on the U.S. EPA’s 2015 guidelines, provides a framework for prioritizing FLW reduction strategies. According to this hierarchy, source reduction is the top preferred solution. As shown in Table 2, a significant portion of studies (61.54%, *n* = 64) do not specify their position within this FRH. Among those that do, industrial uses are the most frequently discussed, accounting for 14.42% (*n* = 15). Source reduction follows closely with 12.5% (*n* = 13). Other categories, such as feeding hungry people (2.88%, *n* = 3), feeding animals (1.92%, *n* = 2), and composting (1.92%, *n* = 2), are less frequently addressed.

The emphasis on industrial uses and source reduction suggests a strong focus on mitigating the environmental impact of FLW through recycling and recovery. However, as Mourad [12] emphasizes, more profound transformations are necessary, concentrating on prevention and tackling the underlying causes of food loss and waste (FLW). Supplying food to those facing hunger needs, which ranks as the second most favored option in this hierarchy, remains insufficiently examined. We advocate for more research on programs/strategies/policies on promoting recovery through donation, redistribution, and building a comprehensive information network to combat FLW with the help of government officials/professionals, program support, and the community. Thirdly, the low prevalence of feeding animals in the U.S. might be due to the strict regulation and policy due to the focus on animal health and related biosecurity practices [58]. Dou et al. [58] indicated that food waste needs to be heated to 100 °C for half an hour before it is suitable for feeding to pigs according to the law, as outlined in the Swine Health Protection Act [73]. Leib et al. [74] reviewed state-level regulations regarding animal feed and reported that twenty states mandate heat processing for both animal and plant waste, whereas twelve states specifically mandate it for animal-derived waste. The restriction and regulation from federal and state levels could affect the adoption of such practice. Similar to the restrictive policy regarding animal feed in the US, the European Union adopted a prohibited policy; suggestions of rethinking those regulations have been proposed [75]. There are still challenges and barriers to promoting feeding animals as a solution. More research is needed to develop strategies that promote food recovery through donation and redistribution, supported by comprehensive information networks and government programs.

Practice evaluation: Based on Moraes et al. [48], practice evaluation divides actions into two primary approaches: prevention and minimization. Prevention includes strategies such as source reduction, aimed at decreasing food loss and waste before it occurs. Conversely, minimization emphasizes measures implemented after food has been manufactured, acquired, or prepared, such as repurposing leftovers or donating surplus to charitable food organizations. The distribution of practice evaluation shows that 61.54% (*n* = 64) of the studies do not evaluate the effectiveness of their proposed solutions. Minimization strategies are the most frequently evaluated, accounting for 25.00% (*n* = 26). Prevention follows with 12.50% (*n* = 13). The mixed category, which includes both prevention and minimization, accounts for 0.96% (*n* = 1).

The observed preference for minimization over prevention may be driven by economic, structural, and policy-related factors. Strong prevention relies on structural changes [12]. Structural changes within food supply chains are often costly, time-intensive, and require sustained coordination among multiple stakeholders, which can slow implementation. Moreover, because supply chains are highly interconnected, changes introduced at one stage can have cascading effects across the entire system, making comprehensive transformation more complex. Given the numerous processes and factors involved, maintaining existing practices is often easier than initiating and sustaining long-term systemic change. In addition, the effectiveness of strong prevention solutions remains unknown [12]. Ultimately, strong prevention is the least emphasized approach, surfacing mainly in fringe social movements or informal individual discussions rather than in official discourse [12]. As a result, stakeholders may prioritize short-term, more readily implementable strategies such as minimization over prevention-oriented approaches that offer longer-term benefits. In addition, political, regulatory, and funding environments may further reinforce this imbalance, as current institutional incentives and policy priorities may favor downstream minimization efforts rather than upstream prevention and source-reduction strategies. In addition, the need to advocate for prevention solutions and educate and encourage government and industry to differentiate weak and strong actions, not only applies to the US setting but also European countries like France, as neither country considered challenging the power dynamics and scale of food commodity chains [12].

While minimization strategies dominate the current literature, prevention remains the most effective strategy for reducing FLW, as it addresses the problem at its source. Papargyropoulou et al. [41] recommend that prevention should be prioritized for its environmental benefits. Future research should focus more on prevention practices, such as source reduction approaches, and on developing methodologies to evaluate these practices’ effectiveness. Encouraging fields within prevention encompass repurposing and byproduct recovery [46], which have the capacity to reduce the ecological impact while retaining it within the human food system.

### 5.4. Machine Learning Assisted Review

As seen in Table 3, the researcher identified themes based on topic modeling results, which are technological solutions for valorizing processing and agricultural waste, FLW drivers and mitigation strategies, cold chain management in produce systems, energy recovery and environmental impacts, postharvest factors influencing fruit quality, shelf life, and loss reduction.

## 6. Conclusions and Limitations

Our scoping review systematically examined the factors, impacts, and potential solutions for FLW in the early stages of the FSC in the U.S. The major contributions of this study are fourfold. First, this review centers on the pre-consumption stages of the food supply chain, providing targeted insights into upstream food loss, which differs substantially from consumer-stage waste. Second, it integrates factors, impacts, and solutions across multiple levels of analysis, addressing limitations of prior reviews that focus on only one dimension and thus overlook the broader system perspective. Third, by synthesizing evidence from the United States—a representative high-income, developed country—this review offers a useful basis for understanding food loss dynamics in Western, industrialized contexts and for comparison with other developed settings. Finally, the study demonstrates the feasibility of incorporating machine learning–assisted methods alongside traditional scoping approaches, highlighting the potential of methodological innovation in evidence synthesis. Overall, this review maps upstream food loss research in the United States and identifies emerging themes and future research needed to support prevention-oriented solutions.

Our findings revealed significant gaps in the literature. Notably, most studies fail to specify macro-level and micro-level factors and impacts, with a predominant focus on environmental consequences while neglecting economic, social, cultural, and political dimensions. Additionally, while there is a strong emphasis on donation, prevention, recovery, and recycling as solutions, critical gaps remain in the exploration of policy and regulatory strategies, as well as education and awareness efforts. The literature also shows a dominant reliance on minimization approaches rather than prevention.

Several studies conducted in Europe have examined food waste reduction. Social factors, particularly consumers’ behaviors and lifestyles, are essential for waste reduction in Europe [76]. Priefer et al. [77] identified broader societal patterns in Europe, including rising wealth, lower food prices, a growing number of single-person households, and higher workforce participation among women. Adjusting social framework conditions can potentially positively influence waste reduction [77]. Future research is encouraged to explore broader societal governance mechanisms such as educational campaigns, nonprofit organization initiatives, and industry-led programs. We also recommend examining communication- and education-based governance instruments, alongside regulatory and economic tools within the U.S. context.

Policy and governance efforts in Europe represent another important set of causes and solutions that can offer valuable insights for the U.S. context. Canali et al. [76] identified that institutional factors, including legislation and policy, are central. Policy interventions are the most promising solutions, such as revising European Union (EU) regulations, eliminating food subsidies, and introducing economic incentives. Priefer et al. [77] argue that policy-level efforts are highly recommended compared to traditional approaches like awareness campaigns, roundtables, networks, and information platforms. A review study on EU legislation identified several key factors influencing food waste generation and management: liability rules for donated foods, date-labeling requirements, the flexibility principle within the EU Hygiene Package, and fiscal regulations [78]. However, previous studies also identified areas for improvement. A review study based on governance analysis stated that current legislation does not effectively guide significant reductions in food waste despite available legal acts. They recommended employing economic policy instruments to address governance challenges [79]. Similarly, Priefer et al. [77] suggested that economic incentives may encourage behavioral change in developed nations, with taxation serving as a good example. In addition, another important policy dimension involves data collection and measurement efforts. Waste statistics were required for EU members every two years, and the improvement of the database has become essential. At the regulatory level, efforts also include revising European marketing standards. Whether the setting of less stringent norms and changing the EU legislation on food safety would reduce food waste remains debated [77], indicating a need for future research in the U.S. context. Several European countries have implemented educational and stakeholder programs. Future studies can examine how institutional coordination, social governance, and policy jointly influence food waste reduction outcomes in the U.S. context.

This review uniquely contributes to the field by highlighting the need for a shift from weak minimization practices to stronger prevention strategies. We advocate more research focused on the origin causes of FLW, the broader impacts of FLW, and the development of comprehensive legislative and regulatory frameworks. Furthermore, we emphasize the importance of education and awareness initiatives to foster behavioral changes. We also call for enhanced cooperation among all the participants in the food chain, from farmers to policymakers, to develop integrated solutions that address FLW more effectively.

This scoping review has several limitations that should be acknowledged. Firstly, the review is limited to the U.S., which affects the applicability of its findings to other regions with different food systems and regulatory frameworks. Secondly, it primarily considers peer-reviewed articles, conference proceedings, and dissertations, which may lead to potential bias by excluding relevant gray literature, government reports, and industry publications. Future researchers can include those for a more comprehensive picture.

Despite these limitations, our scoping review serves as a foundational reference and synthesizes the causes of factors, impacts, and solutions of FLW in the United States, with important implications for both research and practice.

This study reveals significant knowledge gaps within the literature, emphasizing the need for comprehensive research that examines the complete range of factors and consequences linked to food loss and waste (FLW). Future research should focus on areas that have been underexplored, such as cultural and social dimensions, while also developing integrated approaches that blend policy, education, and technological innovation. For practitioners, our review highlights the significance of taking a comprehensive approach to addressing FLW reduction that involves all stakeholders in the FSC and leverages both practical and policy-driven solutions. Many studies did not report the rural or urban status, and this contextual factor can be explored in future research (e.g., national, regional, or local; urban vs. rural). In addition, moving beyond a single-country focus represents an important direction for future research. Comparative syntheses across regions—for example, contrasting U.S. and European evidence bases—could provide additional insights into how structural, policy, and cultural contexts shape food loss research. By filling these gaps, future research and policy can contribute to more effective strategies for reducing food waste and achieving sustainability goals.

## Figures and Tables

**Table 1:** Summary of supply chain phases in FLW.

Previous Studies	Supply Chain Phases in FLW				
	Harvest/ On the Farm/ Agricultural Production/ Slaughter	Post-Harvest Handling and Storage/Slaughter Operations	Processing/ Manufacturing/ Industrial Processing and Packaging	Transportation/ Distribution/Storage	Wholesale, Retail, and Consumer/Public, and Household Consumption
Buzby & Hyman [26], EPA [27]	√		√		√
Conrad [28]	√			√	√
Singh & Singh [29], Porter et al. [13]	√	√	√	√	√
Xue et al. [33]	√	√	√	√	√
Truong et al. [34]	√		√		√
Galford et al. [35]	√		√	√	
Minn [30]	√		√	√	√
Fabi & English [31], Li et al. [32]	√	√	√	√	√

Note. √ indicates that the study addressed food loss and waste (FLW) within the corresponding supply chain phase.

**Table 2:** Donation, prevention, recovery, recycling.

Food Recovery Hierarchy	N	Percentage	Practice Evaluation	N	Percentage
Source Reduction	13	12.50%	Prevention	13	12.50%
Feed Hungry People	3	2.88%	Minimization	26	25.00%
Feed Animals	2	1.92%			
Industrial Uses	15	14.42%			
Composting	2	1.92%			
Mixed	5	4.81%			
			Mixed	1	0.96%
N/A	64	61.54%	N/A	64	61.54%

Note. N/A = not applicable.

**Table 3:** Key themes.

Topic	Keywords	Themes
0	spoilage, hydrothermal, conversion, protein, fuel, products, processing	Technological Solutions for Valorizing Processing and Agricultural Waste
1	unharvested crops, supply chain, recovery, gleaning, organizational interventions, farm, field, strategies	FLW Drivers and Mitigation Strategies
2	fresh, produce, cold, chain, temperature, transportation, safety, practices	Cold Chain Management in Produce Systems
3	energy, biogas, emissions, environmental, water, anaerobic, renewable, sustainable	Energy Recovery and Environmental Impacts
4	fruit, quality, postharvest, storage, shelf, decay, temperature, maturity	Postharvest Factors Influencing Fruit Quality, Shelf Life, and Loss Reduction

## Data Availability

No new data were created or analyzed in this study. Data sharing is not applicable to this article.

## Abbreviations

The following abbreviations are used in this manuscript:

FLW	Food loss and waste
EPA	Environmental Protection Agency
EC	European Commission
UN	United Nations
EU	European Union
USDA	U.S. Department of Agriculture
FSC	Food supply chain
FRH	Food Recovery Hierarchy
AD	Anaerobic Digestion
LDA	Latent Dirichlet Allocation

## References

[1] United Nations, Department of Economic and Social Affairs (2019). Population Division World Population Prospects 2019: Highlights. ST/ESA/SER.A/423. https://population.un.org/wpp/assets/Files/WPP2019_Highlights.pdf.

[2] Vincent H., Jack B., Gagneja K.K. (2020). Food network system secured with blockchain; Zero hunger. Proceedings of the 2020 SoutheastCon, Raleigh, North Carolina, 12–15 March 2020.

[3] Food and Agriculture Organization of the United Nations (FAO) (2020). The State of Food Security and Nutrition in the World 2020. http://www.fao.org/documents/card/en/c/ca9692en.

[4] Coleman-Jensen A., Rabbitt M.P., Gregory C.A., Singh A. (2016). Household Food Security in the United States in 2015.

[5] Bovay J., Zhang W. (2020). A century of profligacy? The measurement and evolution of food waste. Agric. Resour. Econ. Rev..

[6] Hoekstra A.Y., Mekonnen M.M. (2012). The water footprint of humanity. Proc. Natl. Acad. Sci. USA.

[7] Gerber P.J., Steinfeld H., Henderson B., Mottet A., Opio C., Dijkman J., Falcucci A., Tempio G. (2013). Tackling Climate Change Through Livestock: A Global Assessment of Emissions and Mitigation Opportunities.

[8] USDA (2015). Food Waste FAQ’s. https://www.usda.gov/foodwaste/faqs.

[9] United Nations Environment Programme (2024). Food Waste Index Report 2024. https://sdg2advocacyhub.org/wp-content/uploads/2024/03/food_waste_index_report_2024.pdf.

[10] EPA (2021). United States 2030 Food Loss and Waste Reduction Goal. https://www.epa.gov/sustainable-management-food/united-states-2030-food-loss-and-waste-reduction-goal.

[11] Boyer D., Ramaswami A. (2020). Comparing urban food system characteristics and actions in US and Indian cities from a multi-environmental impact perspective: Toward a streamlined approach. J. Ind. Ecol..

[12] Mourad M. (2016). Recycling, recovering and preventing “food waste”: Competing solutions for food systems sustainability in the United States and France. J. Clean. Prod..

[13] Porter S.D., Reay D.S. (2016). Addressing food supply chain and consumption inefficiencies: Potential for climate change mitigation. Reg. Environ. Change.

[14] Sims R., Flammini A., Puri M., Bracco S. (2015). Opportunities for Agri-Food Chains to Become Energy-Smart.

[15] Food and Agriculture Organization of the United Nations 12.3.1 Global Food Losses (Food Loss Index). FAO Sustainable Development Goals Data Portal. https://www.fao.org/sustainable-development-goals-data-portal/data/indicators/1231-global-food-losses.

[16] Minor T., Astill G., Raszap Skorbiansky S., Thornsbury S., Buzby J., Hitaj C., Kantor L., Kuchler F., Ellison B., Mishra A. (2020). Economic Drivers of Food Loss at the Farm and Pre-Retail Sectors: A Look at the Produce Supply Chain in the United States.

[17] Chauhan C., Dhir A., Akram M.U., Salo J. (2021). Food loss and waste in food supply chains. A systematic literature review and framework development approach. J. Clean. Prod..

[18] Dou Z., Ferguson J., Galligan D., Kelly A., Finn S., Giegengack R. (2016). Assessing US food wastage and opportunities for reduction. Glob. Food Secur..

[19] Bellemare M.F., Çakir M., Peterson H.H., Novak L., Rudi J. (2017). On the measurement of food waste. Am. J. Agric. Econ..

[20] Gustavsson J., Cederberg C., Sonesson U., Van Otterdijk R., Meybeck A. (2011). Global Food Losses and Food Waste.

[21] Pinotti K., Luciano A., Ottoboni M., Manoni M., Ferrari L., Marchis D., Tretola M. (2021). Recycling food leftovers in feed as opportunity to increase the sustainability of livestock production. J. Clean. Prod..

[22] Porter S.D., Reay D.S., Higgins P., Bomberg E. (2016). A half-century of production-phase greenhouse gas emissions from food loss & waste in the global food supply chain. Sci. Total Environ..

[23] Nicholes M.J., Quested T.E., Reynolds C., Gillick S., Parry A.D. (2019). Surely you don’t eat parsnip skins? Categorising the edibility of food waste. Resour. Conserv. Recycl..

[24] Lipinski B., World Resources Institute (2016). Food Loss and Waste Accounting and Reporting Standard.

[25] Food and Agriculture Organization of the United Nations (FAO) (2020). Food Loss and Food Waste. https://www.unicef.org/media/72676/file/SOFI-2020-full-report.pdf.

[26] Buzby J., Hyman J. (2012). Total and per capita value of food loss in the United States. Food Policy.

[27] EPA (2016). Advancing Sustainable Materials Management: 2014 Fact Sheet: Assessing Trends in Material Generation, Recycling, Com-Posting, Combustion with Energy Recovery and Landfilling in the United States.

[28] Conrad Z. (2020). Food waste, healthy diets, and environmental sustainability: A guide for nutritionists. Nutr. Today.

[29] Singh A., Singh A. (2022). Microbial degradation and value addition to food and agriculture waste. Curr. Microbiol..

[30] Minn M. (2009). Energy Use in American Food Production. https://michaelminn.net/geography/2009-food-energy/2009-05-11-food-energy.pdf.

[31] Fabi C., English A. (2018). SDG 12.3.1: Global Food Loss Index. http://www.fao.org/3/CA2640EN/ca2640en.pdf.

[32] Li C., Bremer P., Harder M.K., Lee M.S., Parker K., Gaugler E.C., Mirosa M. (2022). A systematic review of food loss and waste in China: Quantity, impacts and mediators. J. Environ. Manag..

[33] Xue L., Liu G., Parfitt J., Liu X., Van Herpen E., Stenmarck Å., O’Connor C., Ostergren K., Cheng S. (2017). Missing food, missing data? A critical review of global food losses and food waste data. Environ. Sci. Technol..

[34] Truong L., Morash D., Liu Y., King A. (2019). Food waste in animal feed with a focus on use for broilers. Int. J. Recycl. Org. Waste Agric..

[35] Galford G.L., Peña O., Sullivan A.K., Nash J., Gurwick N., Pirolli G., Richards M., White J., Wollenberg E. (2020). Agricultural development addresses food loss and waste while reducing greenhouse gas emissions. Sci. Total Environ..

[36] Johnson L.K., Bloom J.D., Dunning R.D., Gunter C.C., Boyette M.D., Creamer N.G. (2019). Farmer harvest decisions and vegetable loss in primary production. Agric. Syst..

[37] Lipinsky B., Hanson C., Lomax J., Kitinoja L., Waite R., Seachinger T. (2013). Reducing Food Loss and Waste.

[38] Parfitt J., Barthel M., Macnaughton S. (2010). Food waste within food supply chains: Quantification and potential for change to 2050. Philos. Trans. R. Soc. B Biol. Sci..

[39] King A., Van de V., Theo G.M. (2013). Chapter 10: Removal of excess cellulose and associated polysaccharides in fruit and vegetable by-products-implication of ruse in feed for monogastric farm animals. Cellulose Fundamental Aspects.

[40] Urbaniak L., Sanchez G., Lee R., Satrio J., Taylor J., Spracklin D. (2021). Value added products from urban organic wastes: A whole systems perspective. Proceedings of the IOP Conference Series: Earth and Environmental Science.

[41] Papargyropoulou E., Wright N., Lozano R., Steinberger J., Padfield R., Ujang Z. (2016). Conceptual framework for the study of food waste generation and prevention in the hospitality sector. Waste Manag..

[42] Venkat K. (2011). The climate change and economic impacts of food waste in the United States. Int. J. Food Syst. Dyn..

[43] Lucera A., Costa C., Conte A., Del Nobile M.A. (2012). Food applications of natural antimicrobial compounds. Front. Microbiol..

[44] Pavlovič I., Knez Ž., Škerget M. (2013). SubcriticalWater—A Perspective ReactionMedia for Biomass Processing to Chemicals: Study on Cellulose Conversion as a Model for Biomass. Chem. Biochem. Eng. Q..

[45] EPA (2015). Sustainable Management of Food. Food Recovery Hierarchy. https://19january2017snapshot.epa.gov/sustainable-management-food/food-recovery-hierarchy_.html.

[46] EPA (2023). From Field to Bin: The Environmental Impacts of U.S. Food Waste Management Pathways (Part 2); EPA/600/R-23/065.

[47] El Bilali H., Berjan S., Ben Hassen T., Memon J.A., Vaško Ž., Allahyari M.S. (2022). Research on food loss and waste in the Western Balkans: A systematic review. Front. Nutr..

[48] Moraes N.V., Lermen F.H., Echeveste M.E.S. (2021). A systematic literature review on food waste/loss prevention and minimization methods. J. Environ. Manag..

[49] Özbük R.M.Y., Coşkun A. (2020). Factors affecting food waste at the downstream entities of the supply chain: A critical review. J. Clean. Prod..

[50] da Costa T.P., Gillespie J., Cama-Moncunill X., Ward S., Condell J., Ramanathan R., Murphy F. (2022). A systematic review of real-time monitoring technologies and its potential application to reduce food loss and waste: Key elements of food supply chains and IoT technologies. Sustainability.

[51] Xu Z., Zhuang X., Ma S. (2025). Machine Learning-Assisted Systematic Review: A Case Study in Learning Analytics. Educ. Sci..

[52] Verma M.K., Yuvaraj M. (2023). AI-based literature reviews: A topic modeling approach. J. Inf. Knowl..

[53] ReFED Who We Are. ReFED. https://refed.org/about/who-we-are/.

[54] Ma S., Zheng X.J., Lu P., Xu Z. (2024). Promoting Upcycled Food: An analysis of social media communication strategies of Upcycled Food Association. Future Foods.

[55] Wusylko C., Weisberg L., Opoku R.A., Abramowitz B., Williams J., Xing W., Vu T., Vu M. (2024). Using machine learning techniques to investigate learner engagement with TikTok media literacy campaigns. J. Res. Technol. Educ..

[56] Python Software Foundation (2024). Python (Version 3.12) [Computer Software]. https://www.python.org/.

[57] Jenkins E.L., Lukose D., Brennan L., Molenaar A., McCaffrey T.A. (2023). Exploring food waste conversations on social media: A sentiment, emotion, and topic analysis of twitter data. Sustainability.

[58] Dou Z., Cochran C., Finn S.M., Galligan D., Goldstein N., O’Donnell T. (2018). Food Loss and Waste: A Paper in the Series on the Need for Agricultural Innovation to Sustainably Feed the World by 2050.

[59] Ceryes C., Heley K., Edwards D., Gao-Rittenberg C., Seifu L., Sohail S.K., Neff R. (2023). “We need a better system”: Maryland crop growers’ perspectives on reducing food loss through donation. J. Agric. Food Syst. Community Dev..

[60] Nourbakhsh S.M., Bai Y., Maia G.D., Ouyang Y., Rodriguez L. (2016). Grain supply chain network design and logistics planning for reducing post-harvest loss. Biosyst. Eng..

[61] Charlebois S., Rankin A., Music J. (2023). Mitigating Food Waste—Are “Best Before” Dates Past Their Due Dates?. Food Prot. Trends.

[62] Thyberg K.L., Tonjes D.J. (2016). Drivers of food waste and their implications for sustainable policy development. Resour. Conserv. Recycl..

[63] Meeker D.L., Meisinger J.L. (2015). Companion animals symposium: Rendered ingredients significantly influence sustainability, quality, and safety of pet food. J. Anim. Sci..

[64] Read Q.D., Muth M.K. (2021). Cost-effectiveness of four food waste interventions: Is food waste reduction a “win–win?”. Resour. Conserv. Recycl..

[65] Kuo J., Dow J. (2017). Biogas production from anaerobic digestion of food waste and relevant air quality implications. J. Air Waste Manag. Assoc..

[66] Stoklosa R.J., Moore C., Latona R.J., Nghiem N.P. (2021). Butyric acid generation by Clostridium tyrobutyricum from low-moisture anhydrous ammonia (LMAA) pretreated sweet sorghum bagasse. Appl. Biochem. Biotechnol..

[67] Aita G.M., Deng F., Cheong D.Y. (2018). Fumaric Acid Fermentation from Lignocellulosic Syrup. ASCPC Conference 2018, New Orleans, LA, USA, 18–21 August 2018.

[68] Dorado C., Cameron R.G., Manthey J.A. (2019). Study of static steam explosion of Citrus sinensis juice processing waste for the isolation of sugars, pectic hydrocolloids, flavonoids, and peel oil. Food Bioprocess Technol..

[69] Yan H., Song M.J., Lee H.Y. (2021). A systematic review of factors affecting food loss and waste and sustainable mitigation strategies: A logistics service providers’ perspective. Sustainability.

[70] Gosliner W., Delaney T., Caldwell S., Lee J.M., Billups N., Floor S. (2020). The Planning, Implementation, and Evaluation of California’s Inaugural Food Waste Prevention Week. J. Public Health Manag. Pract..

[71] Begum T., Follett P.A., Shankar S., Moskovchenko L., Salmieri S., Lacroix M. (2023). Evaluation of bioactive low-density polyethylene (LDPE) nanocomposite films in combined treatment with irradiation on strawberry shelf-life extension. J. Food Sci..

[72] Cattaneo A., Sánchez M.V., Torero M., Vos R. (2021). Reducing food loss and waste: Five challenges for policy and research. Food Policy.

[73] Swine Health Protection Act, H.R. 6593, Congressional Record 96-468 (October 17). Proceedings of the 96th United States Congress.

[74] Leib E.B., Balkus O., Rice C., Maley M., Taneja R., Cheng R., Civita. N., Alvoid T. (2016). Leftovers for livestock: A legal guide for using excess food as animal feed. *The Harvard Food Law and Policy Clinic and the Food Recovery Project at the University of Arkansas School of Law*. https://chlpi.org/wp-content/uploads/2013/12/Leftovers-for-Livestock_A-Legal-Guide_August-2016.pdf.

[75] Shurson G.C., Dierenfeld E.S., Dou Z. (2023). Rules are meant to be broken–Rethinking the regulations on the use of food waste as animal feed. Resour. Conserv. Recycl..

[76] Canali M., Amani P., Aramyan L., Gheoldus M., Moates G., Östergren K., Silvennoinen K., Waldron K., Vittuari M. (2016). Food waste drivers in Europe, from identification to possible interventions. Sustainability.

[77] Priefer C., Jörissen J., Bräutigam K.R. (2016). Food waste prevention in Europe–A cause-driven approach to identify the most relevant leverage points for action. Resour. Conserv. Recycl..

[78] Eriksson M., Giovannini S., Ghosh R.K. (2020). Is there a need for greater integration and shift in policy to tackle food waste? Insights from a review of European Union legislations. SN Appl. Sci..

[79] Garske B., Heyl K., Ekardt F., Weber L.M., Gradzka W. (2023). Challenges of food waste governance: An assessment of European legislation on food waste and recommendations for improvement by economic instruments. Land.

